# The Association Between Dry Eye Disease Symptoms, Digital Device Types, and Screen Distance Among Health Science Students: A Survey‐Based Study

**DOI:** 10.1002/hsr2.71138

**Published:** 2025-10-21

**Authors:** Ragad Allwihan

**Affiliations:** ^1^ College of Science and Health Professions King Saud Bin Abdulaziz University for Health Sciences Jeddah Saudi Arabia; ^2^ King Abdullah International Medical Research Center Jeddah Saudi Arabia; ^3^ Ministry of the National Guard ‐ Health Affairs Jeddah Saudi Arabia

**Keywords:** dry eye disease (DED), electronic devices type, female students, health sciences students, screen distance, symptoms

## Abstract

**Background:**

Electronic devices play a significant role in enhancing the educational experience for students, including those in the health sciences. However, the misuse and overuse of electronic devices can lead to vision health problems. This study aims to investigate the relationship between electronic device types, electronic device screen distance, and dry eye disease (DED) symptoms in female medical students in Saudi Arabia.

**Methods:**

A cross‐sectional survey was conducted on 260 female health sciences students at King Saud Bin Abdulaziz University for Health Sciences (KSUA‐HS), Jeddah, Saudi Arabia, who voluntarily participated online between October 2022 and January 2023. We collected data on electronic device variables and DED symptoms.

**Result:**

Of the 260 participants, 60% (*n* = 157) were aged between 18 and 20; 39% (*n* = 101) were between 21 and 25; and 1% (*n* = 2) were aged 26 and above. Approximately 75.8% of respondents used electronic devices for more than 5 h/day, 65% used tablets, and 51.5% kept a distance of 25 cm from the screen. The reported highest eye symptoms were dryness (8.5%), burning sensation (7.3%), blurring of vision, and watering and tearing of the eyes (6.2%). The difference in the survey parameters was only significantly related to screen distance at 50–100 cm with DED symptoms.

**Conclusion:**

The short screen distance of electronic devices has a significant impact on increasing the DED symptoms among female healthcare students who use electronic devices in Saudi Arabia. This study provides insight into the use of electronic devices to improve diagnostic and preventive strategies for DED symptoms.

## 1 Introduction

Electronic devices play a significant role in enhancing the educational experience of students, including health science students. Using these devices, students can access resources that help them stay updated with the latest research and knowledge [1], and tailor their learning experiences based on their needs [2]. The technology also supports teamwork and collaboration among medical students, which helps them exchange their learning experiences [2]. However, these devices could harm their health. The misuse or overuse of electronic devices can lead to health problems such as backache, headaches, sleep disruption [3], and dry eye disease (DED) [4].

DED, also known as dry eye syndrome, is a chronic disease with a wide range of prevalence. Estimates vary between 5% and 50% of the population globally [5, 6]. It is a “multifactorial disease of the ocular surface, characterized by loss of homeostasis of the tear film and accompanied by ocular symptoms, in which an etiological role is played by the instability and hyperosmolarity of the tear film, inflammation and damage to the ocular surface and neurosensory abnormalities” [7]. Symptoms vary from mild to severe and are linked to itchy eyes, burning eyes, headaches, eye pain, blinking, and sensitivity to light [8].

The increasing prevalence of DED has prompted numerous studies to explore the factors that contribute to the range of DED symptoms and impact various aspects of daily life [9]. Utilizing a comprehensive approach, such as conducting a cross‐sectional survey, to identify different risk factors associated with DED symptoms is essential in mitigating its effects on the population [10]. Age, gender, and smoking are recognized as associated risk factors and have been the subject of various studies [11, 12, 13, 14]. Additionally, several cross‐sectional studies have established a connection between the use of digital screens and ocular diseases, such as DED, among university students [4, 14]. Prolonged screen time on computers and smartphones is significantly associated with dry eye symptoms among university students, with about 90% of them using electronic media for more than 8 h/day [15]. Another study examined computer vision syndrome rates among female undergraduate students in Saudi Arabia and found that DED is one of the common computer vision symptoms, with 51.5% of the study cohort reporting certain computer practices, such as the prolonged use of computers for more than 5 h a day [16]. Another study stated that a sensation of burning eyes (6.7%) and dryness (8.6%) was reported among medical students in Saudi Arabia who used electronic devices [17]. Various studies found that female students were at greater risk of DED symptoms [14, 18, 19]. Smoking habits and duration of electronic device use are considered significant DED symptom risk factors [17].

Previous studies have recommended advising students, including medical students, to use electronic devices in moderation to avoid potentially adverse effects on their visual health [3, 20, 21]. The primary objective of this study was to investigate electronic device types and screen distance as possible risk factors for DED.

## 2 Methodology

### 2.1 Study Design and Population

A validated DED survey (Computer Vision Syndrome Questionnaire (CVS‐Q)) [22] was applied in this study to examine the study objectives. The total number of responses was gathered from a study conducted online between October 2022 and January 2023. This timeframe was chosen to ensure that participants had been experiencing DED symptoms for an extended duration since the onset of COVID‐19, during which the rate of screen time on electronic devices was notably elevated [23]. Only responses from female students were collected, based on the findings of the previous study, which indicated a higher prevalence of DED symptoms among female students as compared to male students [17]. The study was a part of research approved by the Institutional Review Board at King Abdullah International Medical Research Centre (KAIMRC) (IRB/NRJ21J/296/12).

### 2.2 Data Collection

The data were collected by developing an online survey using Microsoft Forms. All the participants signed a written consent form before completing the survey. The data were securely kept, anonymised, and used for research purposes only.

The survey was developed by Mohan et al. [22]. The questions about eye strain symptoms were excluded, and the survey was tweaked slightly to fit the research aims. It was presented in three parts. The first part involved collecting the students' demographic information, including age, educational level, and smoking habits. The second part recorded the students' daily use of electronic devices, including the type of electronic devices they used, screen distance, and screen time duration. The last section recorded common DED symptoms that the students suffered—heaviness, burning, dryness, blinking, watering, redness, sensation, blurring, and itching—as well as the frequency and intensity of these symptoms. This is a self‐reported questionnaire, and the analysis was conducted based on the data we received.

### 2.3 Statistical Analysis

Data analysis was done using Prism version 9. For descriptive statistics, frequency and percentage were computed for the sociodemographic data. The comparison between DED symptoms and intensity levels was presented as frequencies and percentiles. A Shapiro–Wilk test was performed to test the normality of the data.

The linear regression analysis compared students' ages, electronic device types, screen distance, and DED symptoms. A *p* < 0.05 indicates statistical significance. Additionally, the Mann–Whitney–Wilcoxon test was applied to determine the significance values.

The scoring system utilized in this study assigns the following values for intensity and frequency: (*Never* = 1, *Occasionally moderate* = 2, *Occasionally severe *= 3, *Always moderate* = 4, and *Always severe* = 5). If the sum of the scores exceeds 25, then the symptoms of DED are considered positive; conversely, if the sum is ≤ 25, the DED symptoms are deemed to be negative.

## 3 Results

### 3.1 Descriptive Analysis

A total of 260 female students responded to the questionnaires. The demographic data illustrated in Table 1 showed that 60% (*n* = 157) were aged between 18 and 20, and 31.5% (*n* = 82) were in their first academic year. Almost half of the students, 48.5% (*n* = 126), occasionally attended online courses. Regarding smoking, 89% (*n* = 231) have never smoked.

**Table 1:** Description and frequency of demographic information.

Question	Frequency (*n*)	Percentage (%)
Age		
18–20	157	60
21–25	101	38.8
26 and above	2	0.8
Academic year		
1st	82	31.5
2nd	44	16.9
3rd	63	24.2
4th	38	14.6
5th	4	1.5
6th	15	5.8
7th	14	5.4
Attending online courses		
Yes	109	41.9
No	25	9.6
Sometimes	126	48.5
Smoking status		
Never smoked	231	88.8
Occasional smoker	13	5
Heavy smoker	4	1.5
Former smoker	2	0.8
Second‐hand smoker	10	3.8
Electronic device used for online courses		
Computer PC	6	2.3
Laptop	47	18.1
Smartphone	37	14.2
Tablets	170	65.4
Average electronic device's screen distance during studying		
25 cm	134	51.5
50 cm	107	41.2
100 cm	17	6.5
More than 100 cm	2	0.8
Total hours using an electronic device in a day		
< 5 h	35	13.5
5 h and more	225	86.5%

In terms of the type of electronic devices used for online courses, most students (65%, *n* = 170) preferred using tablet devices, such as iPads. Students also varied in how close they kept their electronic devices when studying. Most (51.5%, *n* = 134) kept their devices at a distance of 25 cm. Finally, many students spend a considerable amount of time on electronic devices. A large majority (75.8%, *n* = 197) used their devices for more than 5 h a day.

### 3.2 Comparison Between DED Symptom Frequency and Intensity Level

The comparison between DED symptom frequency and intensity level is shown in Table 2. It is shown that the most common symptoms in the (always to severe) category are a feeling of dryness in the eyes in the past 2 years 8.5% (*n* = 22), a burning sensation in the eyes 7.3% (*n* = 19), a blurring of vision 6.2% (*n* = 16), and watering and tearing of the eyes 6.2% (*n* = 16). The following symptoms showed a less common association with the intensity (never): heaviness in the eyelid 56.5% (*n* = 147); foreign body sensation in the eyes (38.8%, *n* = 101); and/or excessive blinking of eyes (37.7%, *n* = 98).

**Table 2:** Comparison between DED symptoms and intensity level.

Symptoms	Never		Occasionally moderate		Occasionally severe		Always moderate		Always severe	
	Freq.	%	Freq.	%	Freq.	%	Freq.	%	Freq.	%
Have you experienced burning in your eyes in the past 2 years?	27	10.4	152	58.5	40	15.4	22	8.5	19	7.3
Have you experienced itching in your eyes in the past 2 years?	53	20.4	129	49.6	42	16.2	21	8.1	15	5.8
Have you experienced foreign body sensations in your eyes in the past 2 years?	101	38.8	103	39.6	35	13.5	7	2.7	14	5.4
Have you experienced watering/tearing in your eyes in the past 2 years?	73	28.1	110	42.3	25	9.6	36	13.8	16	6.2
Have you experienced excessive blinking of your eyes in the past 2 years?	98	37.7	100	38.5	40	15.4	11	4.2	11	4.2
Have you experienced redness in your eyes in the past 2 years?	61	23.5	138	53.1	35	13.5	16	6.2	10	3.8
Have you experienced pain in your eyes in the past 2 years?	67	25.8	126	48.5	42	16.2	14	5.4	11	4.2
Have you experienced heaviness in eyelids in the past 2 years?	147	56.5	73	28.1	22	8.5	12	4.6	6	2.3
Have you experienced a feeling of dryness in your eyes in the past 2 years?	49	18.8	107	41.2	44	16.9	38	14.6	22	8.5
Have you experienced a blurring of vision in the past 2 years?	68	26.2	112	43.1	33	12.7	31	11.9	16	6.2

### 3.3 Association Analysis Between Student Age, Electronic Device Type, Screen Distance, and the Scoring of DED Symptoms

The linear regression analysis revealed that screen distance (*β* = −0.014, *p* = 0.042) was significantly associated with disease symptom scores. Age (*β* = −0.006, *p* = 0.166) and device type (*β* = 0.005, *p* = 0.501) show a nonsignificant association, where a *p* < 0.05, as shown in Table 3.

**Table 3:** Linear regression coefficients for DED symptom scores by age, device type, and distance.

Variables	Coefficient (slope)	Standard error	*p*
Age	−0.006	0.004	0.166
Device	0.005	0.007	0.501
Screen distance	−0.014	0.007	0.042*

* A *p *< 0.05 indicates a significant difference.

**Figure 1:**
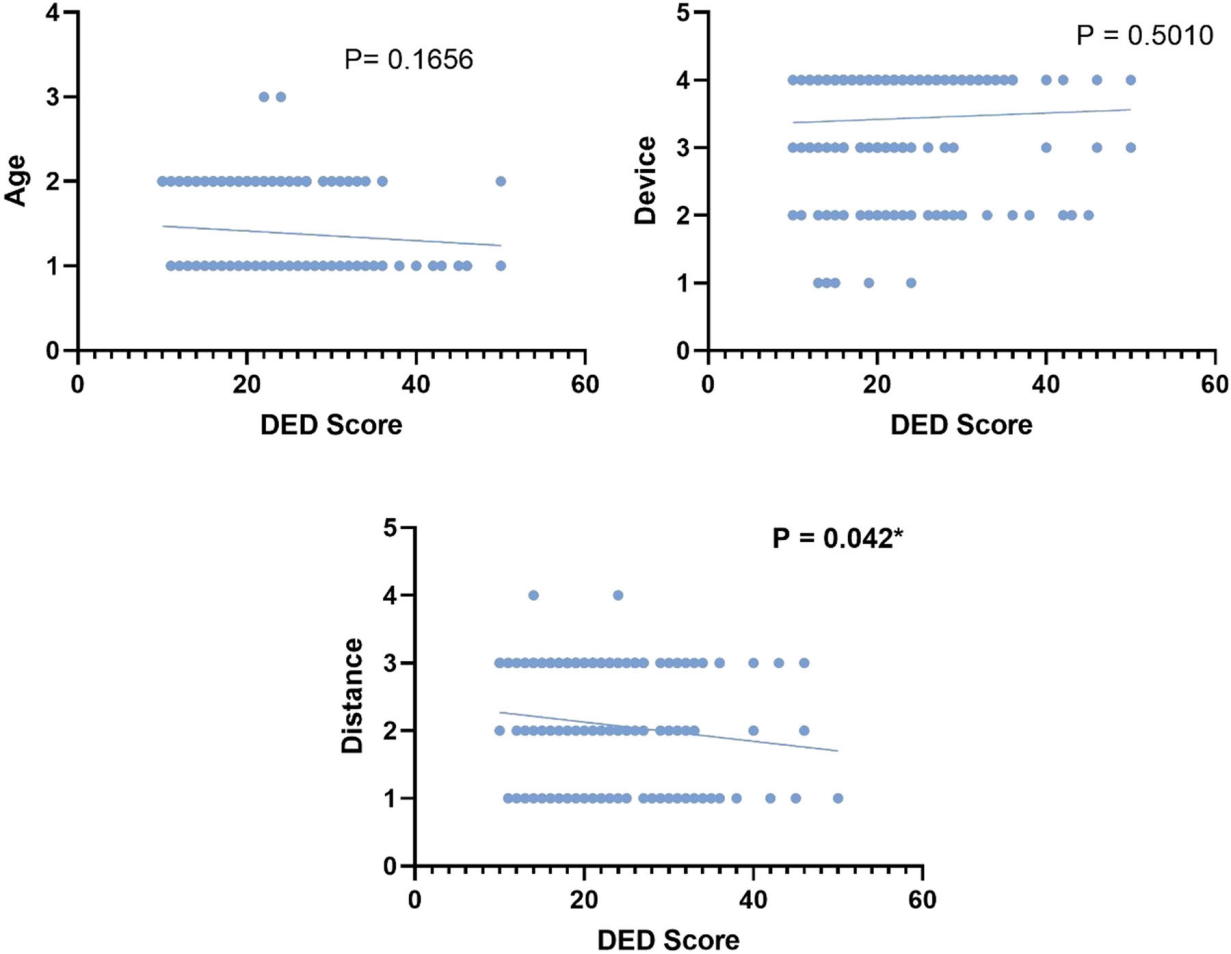
Simple linear regression analysis of DED symptom scores with Age, Device, and Distance. These scatterplots illustrate the associations between DED symptom scores and each predictor variable: Age, Device, and Distance. There is a statistically significant association between Distance and DED symptoms scores (*p* = 0.042*), but no significant effects of Age (*p* = 0.1656) or Device (*p* = 0.5010) on DED scores.

The following scatterplots in Figure 1 illustrate the associations between DED symptom scores and each predictor variable, specifically age, device, and distance. There is a statistically significant association between distance and DED symptom scores (*p* = 0.042*), but no significant effects of age (*p* = 0.1656) or device (*p* = 0.5010) on DED scores.

### 3.4 Comparison Between Screen Distance and the Scoring of DED Symptoms

The Mann–Whitney post hoc test shows that the average of DED symptom scores at 25 cm, 50 cm, 100 cm, and more than 100 cm are equal to 22.04, 21.42, 21.35, and 20.00, respectively.

Notably, the comparison between 50 and 100 cm screen distances reveals a statistically significant difference (*p* = 0.02). There is an observed escalation in symptoms of DED when viewing a screen within the range of 50–100 cm, while other comparative analyses exhibit nonsignificant disparities. This denotes a pronounced impact of these screen distances on DED symptoms, as shown in Table 4.

**Table 4:** The difference between the average scores of DED symptoms at the screen distance during the study.

Screen distance	50 cm	100 cm	More than 100 cm
25 cm	0.98	0.63	0.86
50 cm		0.02*	0.92
100 cm			0.53

* A *p* < 0.05 indicates a significant difference.

## 4 Discussion

This present study investigated the association between DED symptoms, digital device types, and screen distance among female health science students. Its findings support ongoing research on the impact of digital device exposure on eye health in young adults.

Here, the demographic findings indicate that most participants were young adults in their first year of university studies [24]. About half attended online courses occasionally [25]. Furthermore, they expressed a preference for using tablets during online classes [26]. The screen viewing distance was closer than the arm's length distance, which is recommended by the American Academy of Ophthalmology [27]. This aligns with previous studies [16, 28] that reported the majority of students used devices at a distance of < 40 cm and linked this to symptoms of computer vision syndrome during study sessions of over 5 h/day [29]. The majority also reported that they had never smoked [30].

The comparison conducted between the DED symptoms and their frequency and intensity levels among the female students reveals that the intensity of DED symptoms was primarily associated with always severe and most common feelings of dryness in the eyes, a burning sensation, blurring of vision, and watering and tearing of the eyes. This finding aligns with previous studies of university students who used electronic devices, which found that the most frequent symptoms were a burning sensation, eye dryness, blurring sensation, and watery eyes due to behavioral patterns among university students, including intensive interaction with these devices both academically and socially, as well as common physiological behaviors such as alterations in blinking behavior and increased tear evaporation [20, 31].

The association between age and DED symptoms has been the subject of several previous studies. These studies found that ageing is a significant risk factor for DED symptoms, and the prevalence of DED symptoms increases among older populations [11, 12, 32] and medical students [14]. However, this study contradicts the results, which demonstrate any significant difference in DED symptoms between age groups, owing to the narrow age range investigated.

Additionally, this study indicates that there is no significant association between the type of electronic devices used for studying, such as smartphones, computers, and tablets, and an increased risk of DED symptoms among university students. This finding is supported by several studies that have shown screen time, rather than device type, is associated with the development of DED symptoms [33, 34, 35].

The comparison in this study reveals that changing the screen distance between 50 cm and 100 cm significantly affects DED symptoms. According to the American Academy of Ophthalmology, the recommended distance between the screen and the user should be 63.5 cm [27]. This finding supports the previous study's finding that maintaining a distance closer than an arm's length, specifically < 40 cm, is associated with computer vision symptoms, including dry eyes [16, 20, 28]. Several previous studies have shown that the viewing distance of digital devices is influenced by display characteristics which could be a risk factor that causes DED symptoms, such as screen size, brightness, reflections, color combination and font size, human factor characteristics such as arm length and environmental characteristics such as room lighting and the frequency of taking a break from looking at a screen [36, 37].

This study investigates the association between DED symptoms and the type of electronic device and screen distance among female university students. It provides insight into the use of electronic devices to improve diagnostic and preventive strategies for DED symptoms.

This study recommends several strategies with the potential to safeguard ocular health in an increasingly digital world by advocating for users to sustain an appropriate screen viewing distance to enhance ocular health. Additionally, considering the DED symptoms requires a public health approach to promote eye health, such as ensuring regular blinking and adopting the 20‐20‐20 rule.

## 5 Conclusion

Closer electronic device screen distances impact DED symptoms, especially the feeling of dryness, burning sensation, blurring of vision, and tearing of the eyes in female medical students. Students are recommended to maintain safe computing practices and should be encouraged to include electronic device accommodations in their daily lives to prevent the onset of DED. Additional clinical evaluation and investigation ought to be carried out to produce more conclusive evidence.

## 6 Study Limitation

The study was limited as it did not consider some DED symptoms such as double vision, near focusing, halos around objects, light sensitivity, headaches, or deteriorated eyesight. Furthermore, it is important to note that the findings of this cross‐sectional study cannot be generalized to all populations.

## Author Contributions

**Ragad Allwihan:** investigation, writing – original draft, writing – review and editing.

## Ethics Statement

The Institutional Review Board approved the study at King Abdullah International Medical Research Centre (IRB NRJ21J/296/12). All procedures performed in studies involving human participants were in accordance with the ethical standards of institutional and/or research committees and with the 1975 Declaration of Helsinki, as revised in 2013.

## Consent

Informed consent was obtained from the participants.

## Conflicts of Interest

The author declares no conflicts of interest.

## Transparency Statement

The lead author Ragad Allwihan affirms that this manuscript is an honest, accurate, and transparent account of the study being reported; that no important aspects of the study have been omitted; and that any discrepancies from the study as planned (and, if relevant, registered) have been explained.

## Data Availability

The data that support the findings of this study are available from the corresponding author upon reasonable request.
